# Enhancement of Rhodamine B Degradation by Ag Nanoclusters-Loaded g-C_3_N_4_ Nanosheets

**DOI:** 10.3390/polym10060633

**Published:** 2018-06-08

**Authors:** Thi Mai Oanh Le, Thi Hang Lam, Thi Nhung Pham, Tuan Cuong Ngo, Ngoc Diep Lai, Danh Bich Do, Van Minh Nguyen

**Affiliations:** 1 Center for Nano Science and Technology, Faculty of Physics, Hanoi National University of Education, 136 Xuan Thuy Road, Cau Giay District, Hanoi 100000, Vietnam; lemaioanh@gmail.com (T.M.O.L); lamhang289@gmail.com (T.H.L); ptnhungk64hnue@gmail.com (T.N.P); minhsp@gmail.com (V.M.N); 2 Hanoi University of Natural Resources and Environment, Phu Dien Road, North-Tu Liem District, Hanoi 100000, Vietnam; 3 Faculty of Chemistry, Hanoi National University of Education, 136 Xuan Thuy Road, Cau Giay District, Hanoi 100000, Vietnam; cuongnt@hnue.edu.vn; 4 Laboratoire de Photonique Quantiqueet Moléulaire, UMR 8537, Ecole Normale Supérieure de Cachan, Centrale Supélec, CNRS, Université Paris-Saclay, 61 avenue du Président Wilson, 94235 Cachan, France; nlai@lpqm.ens-cachan.fr

**Keywords:** photocatalyst, g-C_3_N_4_ nanosheet, Ag nanoclusters, degradation

## Abstract

In this paper, silver (Ag) nanoclusters-loaded graphitic carbon nitride (g-C_3_N_4_) nanosheets are synthesized and their physical properties as well as photocatalytic activities are systematically investigated by different techniques. The existence of Ag atoms in the form of nanoclusters (NCs) rather than well-crystallized nanoparticles are evidenced by X-ray diffraction patterns, SEM images, and XPS spectra. The deposition of Ag nanoclusters on the surface of g-C_3_N_4_ nanosheets affect the crystal structure and slightly reduce the band gap energy of g-C_3_N_4_. The sharp decrease of photoluminescence intensity indicates that g-C_3_N_4_/Ag heterojunctions successfully prevent the recombination of photo-generated electrons and holes. The photocatalytic activities of as-synthesized photocatalysts are demonstrated through the degradation of rhodamine B (RhB) solutions under Xenon lamp irradiation. It is demonstrated that the photocatalytic activity depends strongly on the molar concentration of Ag^+^ in the starting solution. The g-C_3_N_4_/Ag heterojunctions prepared from 0.01 M of Ag^+^ starting solution exhibit the highest photocatalytic efficiency and allow 100% degradation of RhB after being exposed for 60 min under a Xenon lamp irradiation, which is four times faster than that of pure g-C_3_N_4_ nanosheets.

## 1. Introduction

Graphitic carbon nitride (g-C_3_N_4_), an emerging graphene-like material, has received much attention for its use in numerous applications such as photocatalytic degradation of organic pollutants, conversion of carbon dioxide into hydrocarbons, production of hydrogen and oxygen, etc. The widespread use of the material is owed to its suitable characteristics in crystal structure, surface morphology, and band gap energy as well as its high thermal and chemical stability [1,2]. The band gap of 2.7 eV makes g-C_3_N_4_ an excellent candidate for high conversion efficiency under visible light, particularly in the wavelength range of 400–460 nm [3]. Moreover, g-C_3_N_4_ is inexpensive, abundant, and eco-friendly, making it the most attractive photocatalyst in the field of environmental protection and energy conservation [4]. Nevertheless, photocatalytic performance of pure g-C_3_N_4_ can still be further enhanced in several ways, such as increasing surface area, reducing electron-hole recombination rate, and broadening visible light absorption region to a longer wavelength [5,6].

Various studies have been carried out to improve photocatalytic activities of g-C_3_N_4_ via the above approaches [7,8,9,10,11,12]. Among them, producing heterojunctions between g-C_3_N_4_ and other semiconductors or metal nanoparticles (NPs) has been proven to be an effective solution to separate photo-generated electron-hole pairs and prevent their recombination [9,13,14,15]. Lately, loading noble metal NPs such as Pt [15,16], Au [17,18], Pd [19,20], and Ag [21,22] on the surface of g-C_3_N_4_ nanosheets have been reported as an efficient routine to enhance photocatalytic performance of g-C_3_N_4_. This is because noble metal NPs can act as excellent electron acceptors [23], thus reducing the recombination rate of electron-hole pairs in g-C_3_N_4_ nanosheets and also improving visible-light absorbance [13]. It is important to note that the photocatalytic ability depends strongly on the size of metal NPs. Previous reports have shown that small NPs exhibit strong photocatalytic performance [24,25] due to the high reactivity of unsaturated atoms on the surface of small particles. In particular, when the particle size decreases to subnanometer scale, the NPs are just nanoclusters (NCs) of metal atoms, not crystalline, and they can exhibit interesting photocatalytic abilities [24,26].

In this paper, we report a simple and environmental-friendly approach to synthesize g-C_3_N_4_/Ag heterojunctions in which the surface of g-C_3_N_4_ nanosheets are decorated with Ag NCs to demonstrate the use of the material as an excellent candidate for photodegradation of rhodamine B (RhB). To avoid the leakage of Ag metal into the environment and to determine the optimum loading concentration of Ag NCs on the surface of g-C_3_N_4_ nanosheets, we investigate the dependence of photocatalytic activity of Ag cluster-loaded g-C_3_N_4_ on the concentration of Ag^+^ in the starting solution.

## 2. Experiments 

### 2.1. Materials 

Urea (NH_2_CONH_2_*,* >98%) purchased from Sigma-Aldrich Co. (St. Louis, MO, USA) was used in the polymerization process of g-C_3_N_4_ nanosheets. To synthesize Ag nanoclusters, the precursors were prepared from silver nitrate (AgNO_3_, >99%), a product of Sigma-Aldrich Co. (St. Louis, MO, USA). Ethanol (CH_3_CH_2_OH), a product of Merck (Darmstadt, Germany), was used as a supporting solvent for washing the residual chemical substance contained in the as-synthesized materials. The water used was double distilled to remove unwanted contaminants. All chemical reagents were used as received without further purification.

### 2.2. Synthesis of g-C_3_N_4_

Unlike previous reports [11], in this study, g-C_3_N_4_ was synthesized through urea polymerization in air. In a typical process, 5 g of urea (≥98%, Sigma-Aldrich) was stored in an alumina crucible, which was then covered with an aluminum foil. The crucible was heated to 550 °C in a furnace for 2 h in air. An amount of 0.5 g of bright yellow obtained powder was added into 50 mL of distilled water. The solution was then ultrasonically vibrated for 1 h at room temperature to homogenously disperse g-C_3_N_4_ nanosheets in water.

### 2.3. Synthesis of Ag Nanoclusters-Loaded g-C_3_N_4_

The deposition of Ag NCs on the surface of g-C_3_N_4_ nanosheets was carried out through a simple process without the support of light irradiation. In order to create Ag^+^ starting solution with different Ag^+^ molar concentrations of 0.005 M, 0.007 M, 0.01 M, 0.03 M, 0.05 M, and 0.1 M, respectively, various amounts of AgNO_3_ were added to 50 mL of distilled water. This solution was then poured into the as-prepared, well-dispersed g-C_3_N_4_ nanosheets solution. The suspension was magnetically stirred in the dark for 9 h at a temperature of 90 °C. The grey precipitates were washed three times with distilled water and once with ethanol using an ultrasound vibration and a centrifuge machine at the speed of 4000 rpm. Finally, the obtained powder was dried under vacuum at 60 °C for 10 h before any characterization. 

Ag nanocluster-loaded g-C_3_N_4_ nanosheets synthesized in starting solutions with various Ag^+^ molar concentrations were labeled as gCN/Ag xM, where x refers to the Ag^+^ molar concentrations (x = 0.005, 0.007, 0.01, 0.03, 0.05, 0.1, respectively).

### 2.4. Characterizations

X-ray diffraction (XRD) patterns of Ag cluster-loaded g-C_3_N_4_ nanosheets were recorded by a D8 Advance diffractometer (Bruker, Billerica, MA, USA) using Cu-K_α_ radiation. UnitCell software was used to calculate the lattice parameters of sample from the XRD data. The morphology of as-synthesized samples was carried out by scanning electron microscopy (SEM) technique (S-4800 NIHE microscope, Hitachi, Tokyo, Japan) and transmission electron spectroscopy (TEM) (FEI Tecnai G2 20 TEM). X-ray photoelectron spectroscopy (XPS) was carried out on the Multilab-2000 spectrometer with an Al Kα monochromatized source. Ultraviolet-visible (UV-vis) absorption spectra of the samples were recorded using a Jasco V670 UV-vis spectrophotometer. Fourier transform infrared spectra (FT-IR) were collected on an IR Prestige-21 FT-IR/NIR spectrometer (Shimadzu, Kyoto, Japan). Photoluminescence (PL) spectra were also performed on a fluorescence spectrophotometer (Nanolog iHR 320, Horiba, Kyoto, Japan) using an excitation wavelength of 350 nm.

### 2.5. Investigation of Photocatalytic Activity

In order to assess the photocatalytic performance of as-synthesized g-C_3_N_4_/Ag heterojunctions, the degradation of RhB solution was carried out under the presence of g-C_3_N_4_/Ag heterojunctions using the irradiation of Xenon lamp. In a typical experiment, 0.06 g of Ag cluster-loaded g-C_3_N_4_ nanosheets were dispersed in 30 mL of distilled water using ultrasonic vibrating for 1 h. An amount of 30 mL of RhB 20 ppm aqueous solution was poured into the as-prepared dispersion under magnetic stirring to achieve a final solution of RhB 10 ppm. Before irradiation, the whole solution was magnetically stirred in a dark chamber for 30 min to reach an adsorption-desorption equilibrium state between RhB molecules and Ag cluster-loaded g-C_3_N_4_ nanosheets. An amount of 3 mL of suspension was taken from the reactor every 10 min and centrifuged to remove g-C_3_N_4_. The relative concentration of RhB in the solution as a function of time was evaluated by measuring the intensity of the absorption peak at 554 nm using UV-vis spectrophotometer.

## 3. Results and Discussion

**Figure 1:**
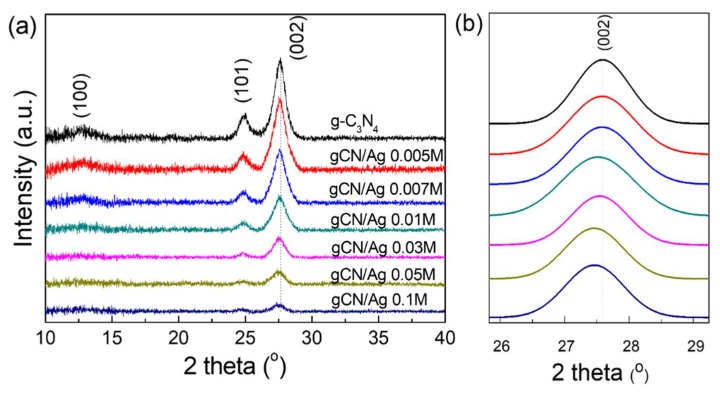
(**a**) XRD diffraction patterns of the as-prepared samples, (**b**) the fitting curves of (002) peak of the as-prepared samples.

The XRD patterns of the as-prepared samples are shown in Figure 1a. It is obvious that pure g-C_3_N_4_ and Ag cluster-loaded g-C_3_N_4_ nanosheets synthesized with different Ag^+^ concentrations exhibited similar X-ray diffraction patterns. As can be seen, pure g-C_3_N_4_ sample exhibited three peaks at around 13.00, 24.93, and 27.65° which were attributed to (100), (101), and (002) diffraction planes, respectively, of the hexagonal phase of graphitic carbon nitride (JCPDS card no. 87-1526). It was found that XRD intensity of Ag cluster-loaded g-C_3_N_4_ samples decreased gradually with increasing Ag^+^ concentrations in the starting solutions. Additionally, the position of (002) peak shifted slightly toward smaller value of 2θ angle. In order to clearly observe the shift of peak position, (002) peaks were normalized to the same intensity and fitted using Gaussian function. Figure 1b shows fitting curves of (002) peak of the as-prepared samples, and the peak position shift is clearly seen. These observations indicate that Ag nanoclusters have a certain influence on the crystal structure of g-C_3_N_4_. However, it is noted that no diffraction peak corresponding to Ag crystal was observed in any of the Ag cluster-loaded g-C_3_N_4_ samples. This may be ascribed to the low concentration of Ag on the surface of g-C_3_N_4_ as well as the amorphous nature of Ag NCs/NPs.

**Figure 2:**
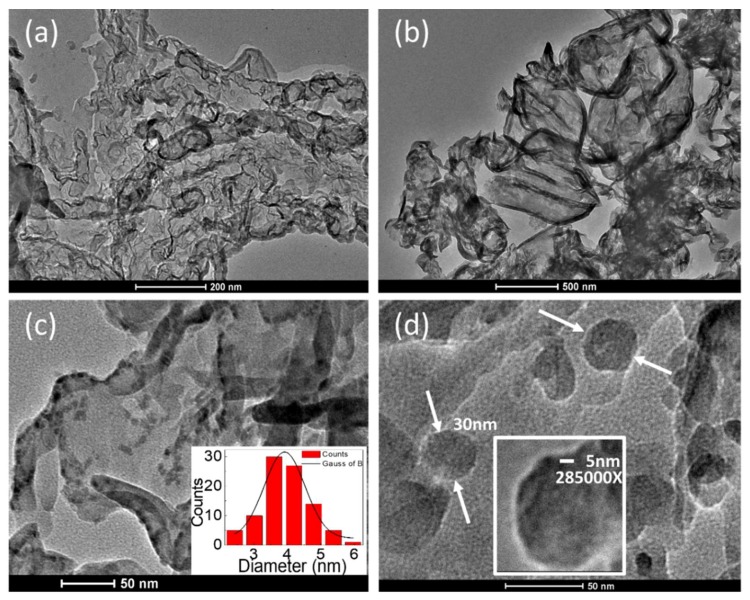
TEM images of the as-prepared (**a**,**b**) pure g-C_3_N_4_ sample, (**c**) g-C_3_N_4_/Ag 0.01 M sample, and (**d**) g-C_3_N_4_/Ag 0.1 M sample. Inset of figure (**c**) shows a histogram of Ag NCs diameters. Inset of figure (**d**) shows the HRTEM image of a single Ag nanoparticle at the magnification of 285000X.

Figure 2 depicts TEM images of the as-prepared samples. As shown in Figure 2a,b, the morphology of pure g-C_3_N_4_ nanosheets is composed of thin layer structures like silk pieces, which is consistent with previous reported results [27,28]. Figure 2c,d demonstrate the presence of dense and homogenous distribution of Ag NCs and Ag NPs decorated on the surface of g-C_3_N_4_ nanosheets in the g-C_3_N_4_/Ag 0.01 M and g-C_3_N_4_/Ag 0.1 M samples. This observation suggests that Ag NPs can act as electron storage tanks, preventing the recombination of photogenerated electron-hole pairs. Inset of Figure 2c displays the histogram of Ag NCs diameters which shows the size of the particles is between 3 and 5 nm (average diameter of 4 nm). This small size means that Ag NPs are more like Ag atom nanoclusters than well-crystallized particles. Figure 2d shows that as the concentration of Ag^+^ increases, the Ag-nanoclusters become nanoparticles with an average size of 30 nm. Additionally, no crystallite formation was found (inset of Figure 2d), which reflects the amorphous nature of NPs. These TEM, HRTEM results partly explain the absence of diffraction peaks of Ag crystal in XRD results.

**Figure 3:**
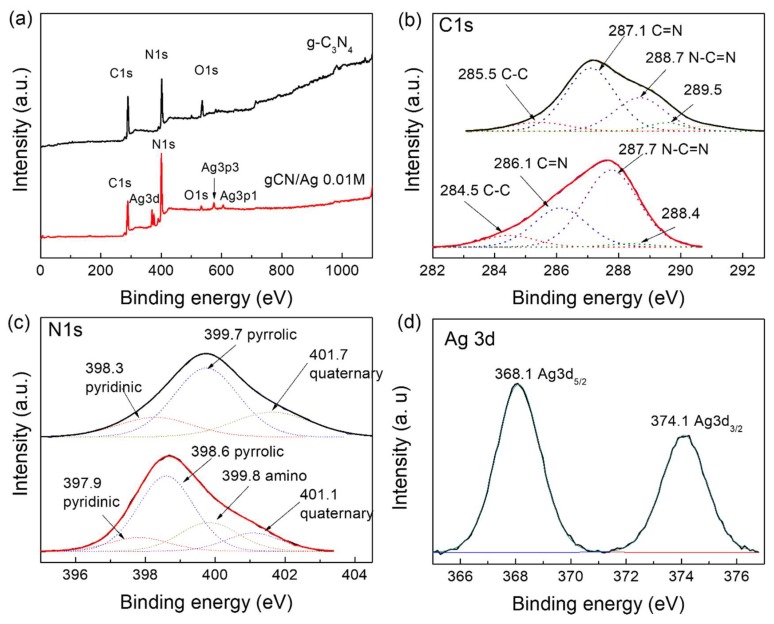
(**a**) XPS survey spectra. XPS spectra of (**b**) C1s, (**c**) N1s and (**d**) Ag3d of pure g-C_3_N_4_ and g-C_3_N_4_/Ag 0.01M samples.

Since the XPS technique can elucidate the chemical compositions and chemical status of different elements on the surface of materials, XPS spectra were carried out to further demonstrate the deposition of Ag nanoclusters on the surface of g-C_3_N_4_ (Figure 3). XPS survey spectra of both pure g-C_3_N_4_ (upper curve) and gCN/Ag 0.01 M (lower curve) samples (Figure 3a) exhibited three peaks at about 287 (C1s), 400 (N1s), and 534 eV (O1s) which were assigned to the presence of C, N, and O elements, respectively, while Ag element was detected in gCN/Ag 0.01 M sample at a binding energy of about 368 eV. Figure 3b shows a zoom-in of the XPS spectra of the C1s peaks for pure g-C_3_N_4_ nanosheets where four secondary peaks with binding energy of 285.5, 287.1, 288.7, and 289.5 eV could be ascribed to: (1) the sp^2^ graphitic carbon (C-C), (2) C=N resulting from defect-containing C atoms, (3) the sp^2^-hybridized C atoms bonded with N atoms in the s-triazine units (N-C=N), and (4) the sp^3^-hybridized carbon atoms (C-(N)_3_) in the g-C_3_N_4_ lattice, respectively [15,29,30]. The high resolution XPS spectra of the N1s peak for pure g-C_3_N_4_ are shown in Figure 3c. This peak was also mainly decomposed into three component peaks centered at about 398.3, 399.7, and 401.7 eV, which corresponded to: (1) pyridinic N, (2) pyrrolic N, and (3) graphitic (quaternary) N, respectively [31,32,33]. In particular, it can be noted that all component peaks for the C1s and N1s peaks shifted to lower energy binding value when Ag NCs deposited on the surface of g-C_3_N_4_, suggesting an electronic interaction between g-C_3_N_4_ and Ag NCs. This observation is similar to those reported by some previous studies [15,34]. Finally, the high resolution XPS spectrum of the Ag3d peak displayed in Figure 3d exhibited two typical peaks at about 368.1 and 374.1 eV, which represented the Ag3d_3/2_ and Ag3d_5/2_ binding energy of the metallic Ag^o^ [35]. The results observed in both XRD patterns and XPS analysis suggest that Ag cluster-loaded g-C_3_N_4_ nanosheets were well synthesized, and the deposition of Ag NCs caused a certain influence on the crystal structure as well as the electronic state of the elements on the surface of g-C_3_N_4_ nanosheets. In other words, there was a certain interaction—or even a good cohesion—between Ag NCs and g-C_3_N_4_ nanosheets, which may become a good channel for photogenerated electron-hole pairs transferring.

**Figure 4:**
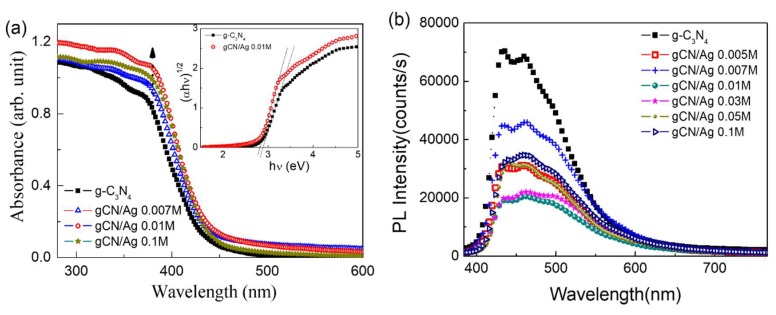
(**a**) UV-vis diffuse reflectance and (**b**) photoluminescence (PL) spectra of as-synthesized samples with different Ag^+^ concentrations.

**Figure 5:**
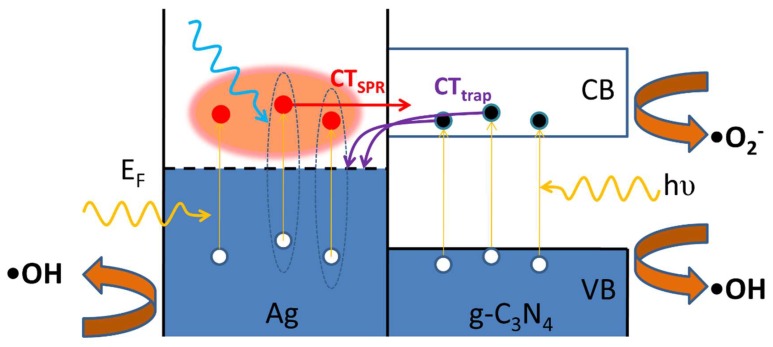
Charge transfer pathways through g-C_3_N_4_/Ag heterojunctions and the generation of active •O_2_^-^ and •OH radicals on g-C_3_N_4_/Ag heterojunctions.

The optical properties of as-synthesized samples were investigated using UV-vis diffused reflectance spectroscopy and photoluminescence (PL) spectroscopy. As can be seen in Figure 4a, pure g-C_3_N_4_ nanosheets exhibited an absorption edge at about 430 nm and the absorption edges of the Ag cluster-loaded g-C_3_N_4_ samples shifted slightly to a longer wavelength, indicating a gradually narrowing band gap. Accordingly, the band gap energy, which was estimated using the Tauc’s plot (the inset of Figure 4a) for indirect semiconductor, decreased from 2.88 eV for pure g-C_3_N_4_ nanosheet to 2.82 eV for gCN/Ag 0.01 M sample. Moreover, Figure 4a also presents an increase of absorbance around 400 nm (the upward solid arrow) for Ag NCs-loaded g-C_3_N_4_ samples, which can be ascribed to absorption due to the surface plasmon resonance (SPR) of Ag NPs [36,37]. These hot SPR electrons in turn can be injected inversely from Ag NPs to g-C_3_N_4_ nanosheets, which is called charge transfer due to SPR effect (CT_SPR_ in Figure 5), enhancing the number of active •O_2_^−^ radicals on the g-C_3_N_4_ surface, while the h^+^ remaining in Ag NPs promotes the production of active •OH radicals on Ag NPs surface [38,39]. On the other hand, SPR electrons can also transfer their energy to stimulate the production of electron-hole pairs in g-C_3_N_4_ nanosheets. Therefore, the high absorbance in Ag cluster-loaded samples due to SPR effect promises a contribution to the degradation of RhB.

Room temperature PL spectra of as-synthesized samples were carried out to investigate the separation behavior of electron-hole pairs through heterojunctions between Ag NPs and g-C_3_N_4_ nanosheets (Figure 4b). It is obvious that pure g-C_3_N_4_ nanosheets presented strong PL intensity, indicating a high recombination rate of electron and hole pairs. By contrast, PL intensity of Ag nanoclusters-loaded g-C_3_N_4_ samples decreased distinctly with increasing Ag^+^ concentration; the lowest PL intensity was obtained by the g-C_3_N_4_/Ag sample having a concentration of 0.01 M. This behavior has been interpreted as the consequence of charge transfer from g-C_3_N_4_ to Ag NCs/NPs due to the energy difference between conduction band (CB) bottom level of g-C_3_N_4_ and Fermi level of Ag NCs/NPs [38]. Therefore, Ag NCs/NPs can act as the “trap” of photogenerated electrons, effectively preventing the recombination of electron-hole pairs in g-C_3_N_4_ nanosheets. For the Ag-NCs of small sizes, the excited energy levels (LUMO) is high and reach the conduction band of the g-C_3_N_4_, therefore facilitating the charge transfers from the g-C_3_N_4_ substrate to the Ag-NCs and quenching the photoluminescence. From the gCN/Ag-0.005 M sample to the gCN/Ag-0.01 M sample, the amount of Ag-NCs increase and we see the decrease in PL intensity, as shown in Figure 4b. As the concentration of Ag^+^ increases, the formed Ag-NCs become larger as shown in Figure 2d. This will result in a reduction in the LUMO level of the Ag-quantum dots, being lower than CB of the g-C_3_N_4_ substrate. The electron transfer from CB of g-C_3_N_4_ to the LUMO level of Ag-NCs will be less supported, as seen in Figure 4b, and the PL intensities of the gCN/Ag-0.03 M, gCN/Ag-0.05 M and gCN/Ag-0.1 M samples are stronger than that of the gCN/Ag-0.01 M sample [40]. The charge transfer due to these “trap” (CT_trap_ in Figure 5) can also make an important contribution to the enhancement of photocatalytic activity.

**Figure 6:**
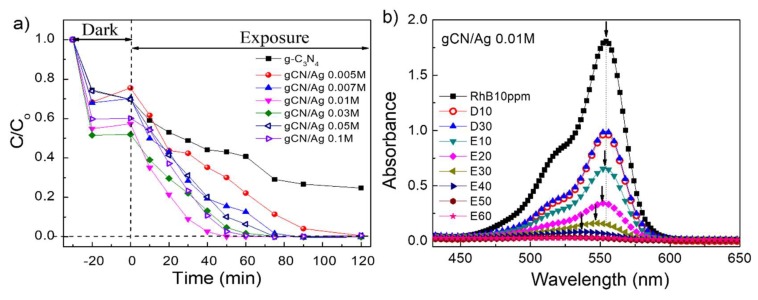
(**a**) Photocatalytic activities of the g-C_3_N_4_ nanosheets and g-C_3_N_4_/Ag heterojunctions with different Ag^+^ concentrations for degradation of RhB under xenon lamp irradiation and (**b**) the sharp decrease of RhB concentration in UV-vis absorption spectrum of gCN/Ag 0.01M sample as a function of time (Dx refers to stirring in dark for x minutes and Ex refers to exposing under Xenon lamp irradiation for x minutes).

The photocatalytic performances of g-C_3_N_4_ and gCN/Ag samples were estimated through the degradation of RhB under irradiation of a Xenon lamp. The degradation rate of RhB was quantified indirectly through the variation of 554 nm absorption peak intensity of RhB solution using a standard curve relating RhB concentration to absorbance. Figure 6a displays the dependence of concentration ratio C/C_o_ on time. The role of adsorption and photocatalysis was assessed separately by stirring 30 min in dark and then 120 min exposed under Xenon lamp irradiation. Obviously, the adsorption process of all samples mostly took place after 10 min of stirring in dark, ensuring that the adsorption-desorption equilibrium state of suspension can be completely achieved after 30 min. Figure 6a shows all Ag NCs/NPs-loaded g-C_3_N_4_ samples displayed a remarkable enhancement of both adsorption and photocatalytic efficiency. While about 25% of RhB still existed after 120 min of irradiation using pure g-C_3_N_4_ nanosheets, some loaded samples such as gCN/Ag 0.01 M, gCN/Ag 0.03 M, and gCN/Ag 0.1 M degraded almost 100% of RhB after just 60 min. The gCN/Ag 0.01 M sample presented strongest photocatalytic activity in RhB decomposing which was evidenced by the largest slope of C/C_o_ curve. The order of samples in which photocatalytic efficiency is increasing is g-C_3_N_4_, gCN/Ag 0.005 M, gCN/Ag 0.007 M, gCN/Ag 0.05 M, gCN/Ag 0.1 M, gCN/Ag 0.03 M, gCN/Ag 0.01 M, and gCN/Ag 0.01 M. Figure 6b exhibits the obvious change of RhB concentration in UV-vis absorption spectra of gCN/Ag 0.01 M heterojunction as a function of time. After 50 min exposure to Xenon lamp irradiation, the 554 nm absorption peak of RhB not only disappeared completely but also shifted from 554 nm to 530 nm, demonstrating the decomposition of conjugated structure of RhB [41]. This enhancement of photocatalytic activity in loaded samples can be adequately explained by the assumption that photogenerated electrons on g-C_3_N_4_ nanosheets effectively transferred to Ag nanoclusters, which reduces the recombination rate of electrons and holes as evidenced by the obvious reduction in PL spectra. In addition, the interpretation of CT_SPR_ as discussed in UV-vis analysis may also contribute to this sharp increase of photocatalytic performance.

## 4. Conclusions

It was demonstrated that g-C_3_N_4_/Ag heterojunctions, in the form of Ag nanoclusters decorated on the surface of g-C_3_N_4_ nanosheets, are an excellent candidate for degradation of the rhodamine B. The Ag NCs/NPs-loaded g-C_3_N_4_ nanosheets were successfully synthesized via a simple approach based mainly on annealing method. Ag NCs/NPs with different size were demonstrated to disperse well on the surface of g-C_3_N_4_ nanosheets and to create bonds with the atoms on the surface of g-C_3_N_4_. The photocatalytic experiment shows that the as-synthesized Ag nanoclusters-loaded g-C_3_N_4_ exhibit much better photocatalytic activity than pure g-C_3_N_4_ nanosheets. It is evident that the photocatalytic efficiency for RhB degradation strongly depends on Ag^+^ concentration in the starting solution and the best photocatalytic efficiency is obtained with 0.01 M of Ag^+^ concentration. The photocatalytic enhancement was demonstrated by the increase of photo-induced electron-hole pairs as well as the effective separation of electrons and holes via g-C_3_N_4_/Ag heterojunctions. These results also provide empirical evidence for the mechanism of quenching the photoluminescence and photocatalysis of the g-C3N4/Ag-NCs or g-C3N4/Ag-NPs composite that has been proposed in previous studies but has not really been confirmed yet.
